# Combined strategy of knowledge‐based rule selection and historical data percentile‐based range determination to improve an autoverification system for clinical chemistry test results

**DOI:** 10.1002/jcla.24233

**Published:** 2022-01-10

**Authors:** Jing Zhu, Hao Wang, Beili Wang, Xiaoke Hao, Wei Cui, Yong Duan, Yi Zhang, Liang Ming, Yingchun Zhou, Haitao Ding, Hongling Ou, Weiwei Lin, Liu Lu, Yuanjiang Shang, Yong Yang, Xianming Liang, Jiangtao Ma, Wenhua Sun, Te Chen, Guang Han, Meng Han, Weiting Yu, Baishen Pan, Wei Guo

**Affiliations:** ^1^ Department of Laboratory Medicine Zhongshan Hospital Fudan University Shanghai China; ^2^ Department of Laboratory Medicine, Xiamen Branch Zhongshan Hospital Fudan University Shanghai China; ^3^ Department of Laboratory Medicine, Wusong Branch Zhongshan Hospital Fudan University Shanghai China; ^4^ Xijing Hospital Xi'an China; ^5^ Cancer Hospital Chinese Academy of Medical Sciences Beijing China; ^6^ First Affiliated Hospital of Kunming Medical University Kunming China; ^7^ Qilu Hospital of Shandong University Jinan China; ^8^ The First Affiliated Hospital of Zhengzhou University Zhengzhou China; ^9^ The First Affiliated Hospital of Guangzhou University of Chinese Medicine Guangzhou China; ^10^ Inner Mongolia People’s Hospital Huhhot China; ^11^ Chinese People’s Liberation Army Rocket General Hospital Beijing China; ^12^ Renji Hospital Shanghai Jiaotong University School of Medicine Shanghai China; ^13^ Shanghai Dongfang Hospital Shanghai China; ^14^ Tenth Peoples Hospital of Tongji University Shanghai China; ^15^ The Second Affiliated Hospital of Soochow University Suzhou China; ^16^ Zhongshan Hospital Xiamen University Xiamen China; ^17^ Shenzhen People’s Hospital Shenzhen China; ^18^ Shanghai Songjiang District Central Hospital Shanghai China; ^19^ The Hospital Group of The First Affiliated Hospital of Chongqing Medical University Chongqing China; ^20^ Guangdong Provincial TCM Hospital Guangzhou China; ^21^ Tianjin First Central Hospital Tianjin China; ^22^ Tongji Medical College Huazhong University of Science and Technology Wuhan China

**Keywords:** autoverification system, clinical chemistry test report, efficiency, historical data percentile‐based, knowledge‐based

## Abstract

**Background:**

Current autoverification, which is only knowledge‐based, has low efficiency. Regular historical data analysis may improve autoverification range determination. We attempted to enhance autoverification by selecting autoverification rules by knowledge and ranges from historical data. This new system was compared with the original knowledge‐based system.

**Methods:**

New types of rules, extreme values, and consistency checks were added and the autoverification workflow was rearranged to construct a framework. Criteria for creating rules for extreme value ranges, limit checks, consistency checks, and delta checks were determined by analyzing historical Zhongshan laboratory data. The new system's effectiveness was evaluated using pooled data from 20 centers. Efficiency improvement was assessed by a multicenter process.

**Results:**

Effectiveness was evaluated by the true positive rate, true negative rate, and overall consistency rate, as compared to manual verification, which were 77.55%, 78.53%, and 78.3%, respectively for the new system. The original overall consistency rate was 56.2%. The new pass rates, indicating efficiency, were increased by 19%‒51% among hospitals. Further customization using individualized data increased this rate.

**Conclusions:**

The improved system showed a comparable effectiveness and markedly increased efficiency. This transferable system could be further improved and popularized by utilizing historical data from each hospital.

## Graphical abstract

- The clinical chemistry report results are evaluated following the process flow.
- The rules are selected by medical laboratory experts.
- The criteria for each rule are determined using historical data.

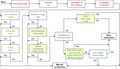

## 1 INTRODUCTION

Medical laboratory technologists usually have to make a quick judgment on a vast number of reports in a short period of time. However, with the increasing number of test specimens, the traditional manual auditing mode has drawbacks under high pressure. Technologists inevitably become fatigued, and mistakes may occur. Therefore, an autoverification (automated result verification) system is needed to reduce the proportion of manual audits, improve turnaround time, and identify false reports.^1^ Autoverification is the automated action of a computer system, related to the release of test results to patients’ medical records, using rules and criteria established, documented, and tested by the medical staff of the laboratory.^2^ An autoverification system can ensure that each result is verified by a sophisticated and consistent verification process. It also intercepts potentially false results and provide necessary warnings to medical laboratory technologists. After the establishment and implementation of an autoverification system, it should be optimized and validated constantly to achieve greater performance.

In Zhongshan laboratory, a clinical chemistry autoverification system was established and implemented in 2012, according to Valdiguié’s study and the Clinical & Laboratory Standards Institute (CLSI) Autoverification of Clinical Laboratory Test Results, Approved Guideline (AUTO 10‐A).^2^, ^3^ At that time, limited numbers of test specimens were processed. The knowledge‐based autoverification ranges, set by experienced clinical pathologists and medical laboratory technologists, were strict, with a true negative rate of 69%. Consequently, laboratory technologists expended much effort to review false positive reports. Currently, the increasing numbers of test specimens have imposed an added reviewing workload, bringing challenges to Zhongshan laboratory and other medical laboratories across China.^4^, ^5^, ^6^, ^7^ Thus, there is a need to replace the original knowledge‐based‐only system with a system with comparable effectiveness and higher efficiency.

The experience of laboratory technologists is based on their history of auditing reports. Due to the dietary changes, upgrading of laboratory technology, etc., data generated in a laboratory are dynamic. Rather than relying on human experience, a regular statistical analysis of periodic historical data may be a better way to determine the autoverification ranges. In this study, we explored the feasibility and practicality of using knowledge to select autoverification rules and historical data to determine autoverification ranges. The system established in this way was compared with the original knowledge‐based‐only system to assess whether it was an improvement.

## 2 MATERIALS AND METHODS

### 2.1 Original autoverification system

The original autoverification system of Zhongshan Hospital was designed and established according to Valdiguié’s study and the CLSI AUTO 10‐A document in 2012.^2^, ^3^ The system covered 60 items (tests), including liver function, kidney function, lipids, immunoglobulins, electrolytes, enzymes, and hemolysis, icterus, and lipemia indexes (HIL indexes). It included 359 rules (Table S1). These rules were divided into six types: instrument analytical range (120 rules), instrument alarm (107 rules), critical value (8 rules), logic among tests (4 rules), limit checks (60 rules), and delta checks (60 rules). The tests covered by critical value rules were calcium (CA), sodium (NA), potassium (K), and fasting glucose (GLU), whereas the tests covered by logic rules included total bilirubin (TBIL), direct bilirubin (DBIL), total protein (TP), albumin (ALB), total cholesterol (TC), high density lipoprotein (HDL), low density lipoprotein (LDL), NA, chlorides (CL), total carbon dioxide (TCO2), and anion gap (AG). In the autoverification workflow, the autoverified report should not violate any of the 359 rules (Figure S1).

### 2.2 Modification of autoverification system framework

Two new types of rules, ie, extreme value (60 rules) and consistency check (9 rules) rules were added in the improved system; thus, the new system comprised 428 rules in total (Table S1). The tests covered by consistency check rules included immunoglobulin G (IgG), immunoglobulin A (IgA), immunoglobulin M (IgM), immunoglobulins of the kappa light chain‐type (KAP), immunoglobulins of the lambda light chain‐type (LAM), triglycerides (TG), lipemia (L) index, creatinine (CRE), urea (UREA), alanine aminotransferase (ALT), aspartate aminotransferase (AST), TBIL, DBIL, alkaline phosphatase (ALP), L‐γ‐glutamyl transferase (γ‐GT), NA, CL, CA, and inorganic phosphorus (P).

**FIGURE 1:**
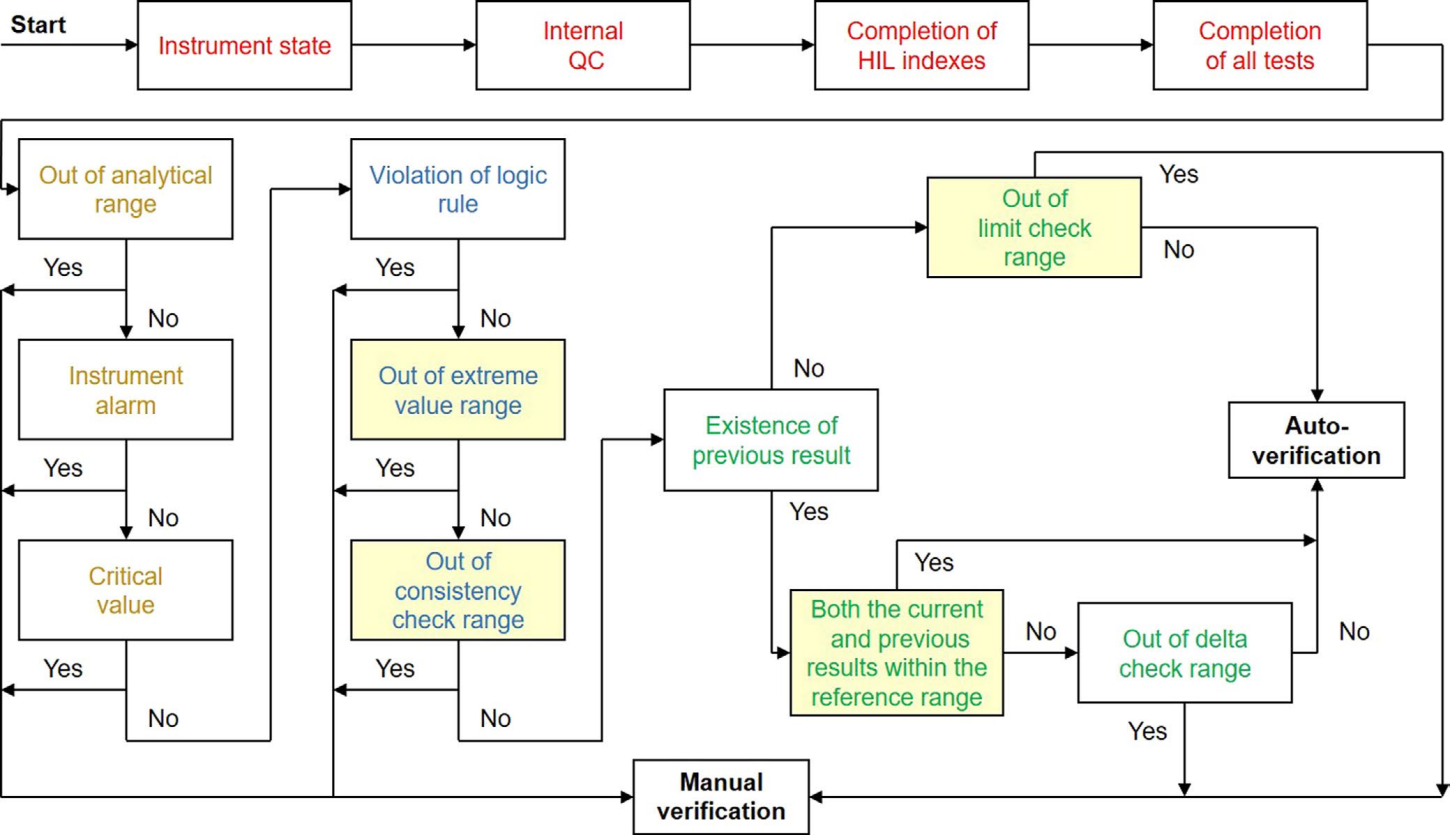
Framework of the improved clinical chemistry autoverification system. The workflow included 4 steps, the readiness of the test results (red text), the instrumental preset rules (yellow text), the laboratory established rules (blue text), and the comparison (with historical results) rules (green text). The rules in the filled boxes (light yellow) were newly added or rearranged. The autoverification would not be initiated until all the results of a report were ready. If any rule was violated, the report could not be autoverified and it would be transferred to manual verification. QC, quality control

The improved system also collected data for a report (patient information, HIL indexes, test results, sample information, and instrument flags) and worked following the modified framework (Figure 1). In this framework, if there was a previous result, the report would not undergo a limit check. If both the current and previous results were within the reference range, a delta check was not needed. The priorities of the rules in the system indicated their importance. This modified framework was defined as the Zhongshan framework.

### 2.3 Determination of the range of each autoverification rule

In the original system, the ranges of limit check rules were determined by knowledge of experienced technologists. The ranges of delta check rules were set according to the Clinical Laboratory Improvement Amendments of 1988 or the local government‐published total errors. During the improvement of the system, 113,956 routine clinical reports for the Han Chinese individuals were generated from February 1, 2017, to May 1, 2017, at Zhongshan laboratory; these were extracted from the laboratory information system (LIS). The frequency distribution of each item, each consistency check, and each delta check change were analyzed using the historical clinical report data. The ranges of extreme value rules and limit check rules were determined based on the frequency distributions of each item. The ranges of consistency check rules and delta check rules were set based on the frequency distributions of each consistency check and each delta check change, respectively. Generally, according to the frequency distributions, the reports intercepted by any individual rule should be fewer than 10%, and the ranges of extreme value rules should be wider than those of limit check rules. The finalized ranges were defined as the Zhongshan criteria.

### 2.4 Validation of the improved autoverification system

The improved autoverification system contained the Zhongshan framework and the Zhongshan criteria. The system was validated according to the guidance documents of the International Organization for Standardization (ISO), College of American Pathologists, and CLSI.^2^, ^8^, ^9^ The validation process involved 20 laboratories throughout China (Table S1). All these laboratories utilized the same instruments and reagents. They were all accredited by the governmental authority and qualified by the National External Quality Assessment of China. In addition, 90% of them had achieved ISO 15189 accreditation, which is not mandatory in China. In all these laboratories, IgG, IgA, IgM, immunoglobulin E (IgE), complement C3 (C3), complement C4 (C4), and homocysteine (HCY) were analyzed using a Hitachi modular P analyzer (Hitachi Ltd.) with DiaSys reagents (Shanghai, China), while iron and unsaturated iron binding capacity (UIBC) were analyzed with the same analyzer, using Wako reagents (Osaka, Japan). The other clinical chemistry tests were conducted on a Roche Cobas 8000 modular analyzer series using Roche reagents (Roche). The test results were transferred to LIS using Roche middleware IT3000.

The routine clinical reports generated for the Han Chinese population were collected from the participating hospitals, totaling 2,246,697 (Table S1). Of these, 20,996 reports (ca. 1% of the total reports) were randomly selected and manually verified by an expert panel consisting of members from all 20 hospitals. The manual verification was achieved upon consensus of at least two medical laboratory technologists with rich experience in clinical chemistry report verification and an approval by the expert panel. Each manually verified report was judged by the improved (Zhongshan framework and Zhongshan criteria), intermediate (original framework and Zhongshan criteria), and original (original framework and original knowledge‐based criteria) autoverification systems, separately. Comparison between the autoverification and manual verification results showed the effectiveness of each system (Figure S1). To determine whether the improvements reduced the proportion of manual audits required and increased efficiency, all 2,246,697 reports were analyzed by both the original system and the improved system, and the efficiencies (pass rates) of the two systems were compared for the 20 laboratories.

### 2.5 Further improvement of the autoverification system

For the laboratory with the lowest pass rate for the improved system, the autoverification system was further improved by determining the ranges of all 60 delta check rules, using its own historical data rather than the Zhongshan data, and the same percentiles as set in the Zhongshan criteria. The efficiency (pass rate) of this laboratory was then re‐evaluated using the further improved system consisting of the Zhongshan framework and partially customized criteria.

### 2.6 Ethics approval

This study was approved by the Research Ethics Committee at each medical center in agreement with the World Medical Association's Declaration of Helsinki.

## 3 RESULTS

### 3.1 Logic and consistency check rules

**FIGURE 2:**
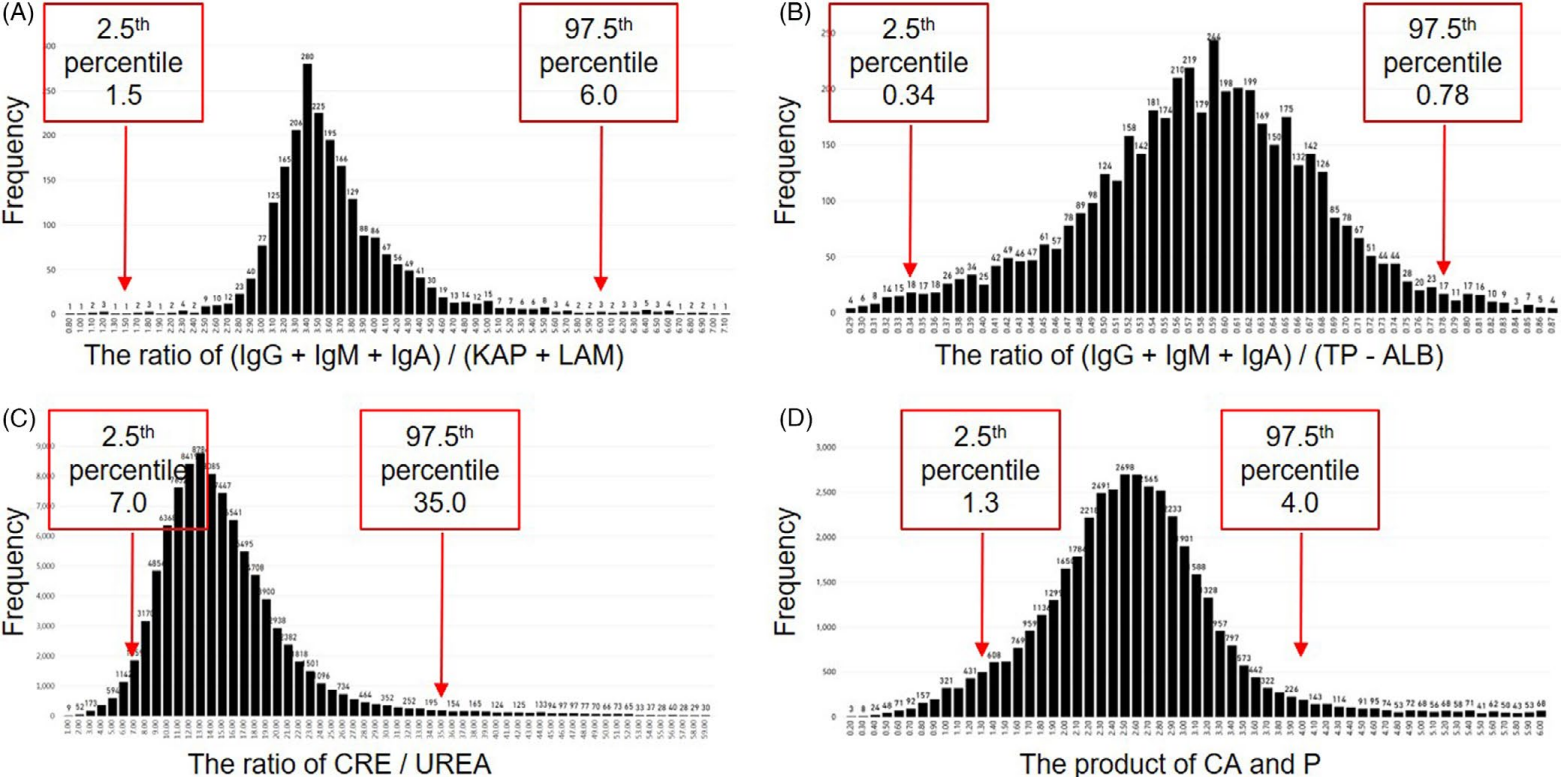
Frequency distributions of some consistency check values. The values we showed include the ratio of (IgG +IgM + IgA)/(KAP +LAM) (A), the ratio of (IgG +IgM + IgA)/(TP ‐ ALB) (B), the ratio of CRE/UREA (C) and the product of CA and P (D). The 2.5th and 97.5th percentiles were used to establish the consistency check rules

The tests in a report reflect the condition of the same patient from different angles; hence, their results are internally related. Some minor problems, such as sample aspiration error, drug interference, substrate depletion, or a high‐dose hook effect, may not cause extremely abnormal results but will have violated the internal relations. Thus, consistency check rules were newly added to identify these violations. The 9 consistency check rules included (IgG + IgM + IgA)/(KAP + LAM), (IgG + IgM + IgA)/(TP‐ALB), CRE/UREA, product of CA and P, whose frequency distributions of historical data are shown as representative data in Figure 2. The distributions of the four consistency check rules were convergent, and each curve had only one spike, which supported the existence of inter‐test relations. The 2.5th–97.5th percentiles of each distribution were used as the autoverification ranges for the four consistency check rules (Figure 2).

In addition, the logic rules reflect the known logical relations among tests. The autoverification range of each logic or consistency check rule is shown in Table 1.

**TABLE 1:** Logic and consistency check rules among tests. The logic rules were established according to the biological rationales, while the consistency check rules were established by analyzing the historical data

No	Rule	Autoverification range
Logic		
1	TBIL − DBIL	>0
2	TP − ALB	>0
3	TC − HDL − LDL	>0
4	NA − CL − TCO2 − AG	=0
Consistency check		
1	(IgG + IgA + IgM)/(KAP + LAM)	1.5–6.0
2	(IgG + IgA + IgM)/(TP − ALB)	0.34–0.78
3	TG/L Index	0.01–
4	CRE/UREA	7.0–35.0
5	If ALT <50, ALT/AST	0.19–1.55
	If ALT ≥50, ALT/AST	0.39–2.99
6	TBIL/DBIL	1.5–4.2
7	ALP/γ‐GT	0.5–6.1
8	NA /CL	1.33–1.46
9	CA × P	1.3–4.0

### 3.2 Extreme value ranges, limit check ranges, and delta check ranges

Similar to the consistency check rules, the frequency distribution of each item was analyzed using the historical data. The 0.1th–99.9th percentile and the 2.5th–97.5th percentile of each distribution were used as the extreme value range and limit check range, respectively (Table S2).

**FIGURE 3:**
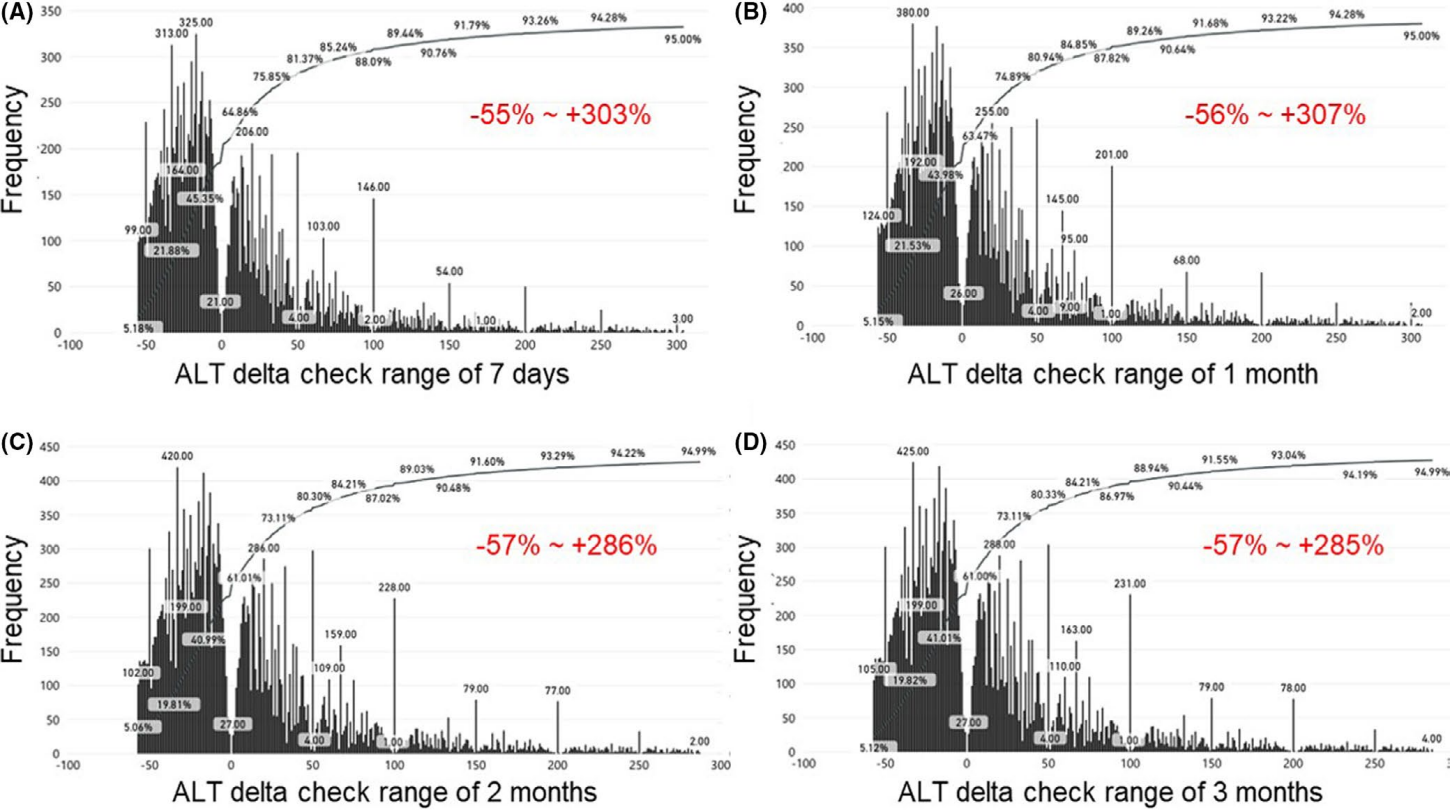
Frequency distributions of ALT delta check within different time periods. The delta check of ALT was measured using the latest result within 7 days (A), 1 month (B), 2 months (C), and 3 months (D). The delta check ranges (the 5th to 95th percentile) were shown in red

Since two consecutive results for the same patient generally do not vary markedly, the delta check also helps to judge if the current result is plausible. The current results from the historical data were compared with the previous results within different time periods. As a representative result, the frequency distributions of the ALT delta check are shown in Figure 3 (A, within 7 days; B, within 1 month; C, within 2 months; D, within 3 months). If the current results equaled previous results, they were not included in the frequency distribution analysis. For all four time periods, the 5th to 95th percentiles of the distributions were similar. Considering that out‐patients are not tested frequently, the delta check range was determined using a comparison within 3 months. The determined extreme value ranges, limit check ranges, and delta check ranges for 11 tests of liver functions, including TBIL, DBIL, TP, ALB, ALT, AST, ALP, γ‐GT, lactate dehydrogenase (LDH), total bile acid (TBA), and prealbumin (PA), are shown in Table 2.

**TABLE 2:** Extreme value ranges, limit check ranges, and delta check ranges for the tests of liver functions. The extreme value ranges (the 0.1th to 99.9th percentile) and limit check ranges (the 2.5th to 97.5th percentile) were determined using routine laboratory results. The determination of delta check ranges is described in Figure 3

No	Test	Unit	Extreme value range	Limit check range	Delta check range
1	TBIL	µmol/L	1.8–409.2	3.7–45.4	−50% – +132%
2	DBIL	µmol/L	0.3–341.9	1.1–27.2	−52% – +159%
3	TP	g/L	38–89	51–79	−18% – +15%
4	ALB	g/L	19–51	29–48	−18% – +17%
5	ALT	U/L	2–1159	6–152	−57% – +285%
6	AST	U/L	6–1184	11–122	−63% – +248%
7	ALP	U/L	24–1057	39–267	−31% – +50%
8	γ‐GT	U/L	6.4–41.9	10–28	−42% – +131%
9	LDH	U/L	5–2695	131–518	−34% – +78%
10	TBA	µmol/L	0.1–367.7	1–57	−88% – +416%
11	PA	g/L	0.04–0.55	0.08–0.39	−42% – +48%

### 3.3 The effectiveness of the improved autoverification system

The effectiveness of the improved system, according to the rule ranges based on Zhongshan Hospital data, was evaluated with 20,996 reports, which were randomly selected from the 20hospitals (Table S2). These reports were judged by both the expert panel (manual verification) and the autoverification system (Table 3; Figure S2).

**TABLE 3:** Evaluation of the autoverification system. The evaluation was achieved by comparing the judgments between autoverification and manual verification. The original system (original framework and knowledge‐based criteria), intermediate system (original framework and historical data percentile‐based Zhongshan criteria), and improved system (Zhongshan framework and Zhongshan criteria) were evaluated

	Manual verification		True positive rate	True negative rate	False positive rate	False negative rate	Negative predictive value	Positive predictive value	Overall consistency rate	Overall pass rate
	Fail	Pass								
Auto‐verification (improved)										
Fail	1366	4119	75.55%	78.53%	21.47%	24.45%	97.15%	24.90%	78.3%	73.9%
Pass	442	15069								
Auto‐verification (intermediate)										
Fail	1396	4780	77.21%	75.09%	24.91%	22.79%	97.22%	22.60%	75.3%	70.6%
Pass	412	14408								
Auto verification (original)										
Fail	1605	8997	88.77%	53.11%	46.89%	11.23%	98.05%	15.14%	56.2%	49.5%
Pass	203	10191								

Our improvement strategy included knowledge‐based rule selection (Zhongshan framework) and historical data percentile‐based range determining (Zhongshan criteria). Hence, three autoverification (improved, intermediate, and original) systems were analyzed for evaluating effectiveness, to show the separate contributions of the Zhongshan framework and Zhongshan criteria. In the original system, the true positive rate (sensitivity) was 88.77%, the true negative rate (specificity) was 53.11%, and the overall consistency rate (between manual verification and autoverification) was 56.2% (Table 3). In the intermediate system, the true positive rate was 77.21%, the true negative rate was 75.09%, and the overall consistency rate was 75.3%, indicating that the historical data percentile‐based range determination improved specificity and overall effectiveness. The positive predictive rate was also improved from 15.14% to 22.60%. Similarly, the true negative rate (78.53%), positive predictive rate (24.90%), and overall consistency rate (78.3%) in the improved system showed that knowledge‐based rule selection further improved specificity and overall effectiveness. Furthermore, the increased overall pass rates suggested that the efficiency was also improved by the new system.

### 3.4 The pass rates of 20 laboratories

**FIGURE 4:**
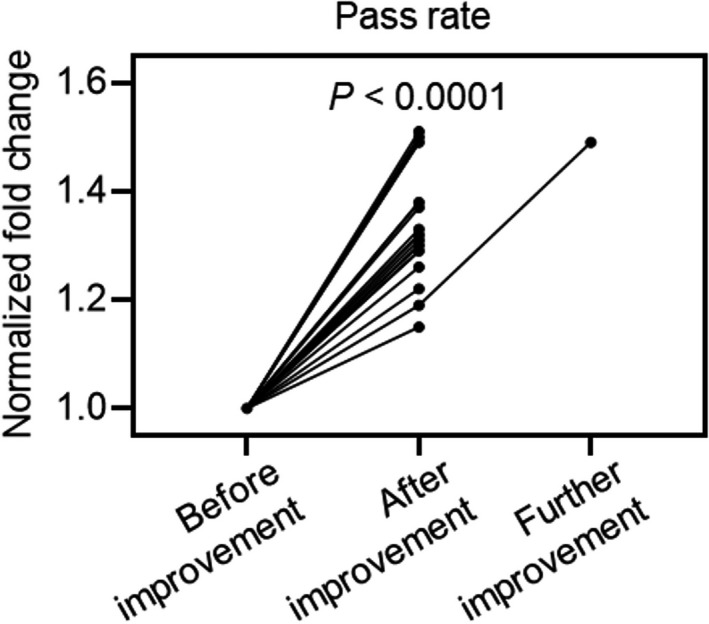
Normalized fold changes of pass rates among 20 laboratories. The pass rates were normalized to that of the original (before improvement) autoverification system at each laboratory. Each dot represented a normalized fold change of pass rate. The dots from the same hospital were linked. For the laboratory (No. 20) with the lowest pass rate, the autoverification system was further improved. The *P* value from paired student's t test between original and improved systems was shown

The purpose of the autoverification system is to emancipate laboratory technologists from needing to perform a manual audit. Its efficiency may be assessed by the pass rate. The reports from each of the 20 laboratories were analyzed by the original and improved autoverification systems (Table S2). The improved system increased the pass rate among the 20 laboratories by 19%‒51%, and the increases were statistically significant, indicating that efficiency was markedly enhanced by the improved system (Figure 4; Figure S3).

For the laboratory (No. 20) with the lowest pass rate in the improved system (43%, Figure S3), we analyzed the intercepted false positive reports and found that some of these reports violated delta check rules. Considering that the Zhongshan criteria, which were possibly not suitable for laboratory No. 20, were used as the ranges for these rules, we customized the ranges of all 60 delta check rules using this laboratory's own historical data. The autoverification system using the Zhongshan framework and partially customized range criteria for this laboratory resulted in an increased pass rate of 49% (compared to 19% without customization), suggesting that, for each individual laboratory, an autoverification system consisting of the Zhongshan framework and individualized criteria using its own historical data may be a better option (Figure 4).

## 4 DISCUSSION

In this study, we attempted to enhance autoverification systems by selecting autoverification rules based on knowledge and ranges based on historical laboratory data. Compared with the original knowledge‐based system, the improved system showed a comparable effectiveness and markedly increased efficiency. This system was transferable across laboratories and could be further improved and popularized by utilizing historical data from the individual laboratory.

In manual verification or knowledge‐based autoverification, the report is typically considered to be abnormal (and intercepted), because a test result is too high or too low and beyond a limit check range. However, test errors caused by minor problems, such as substrate depletion or the hook effect, are not always obvious and are thus easily ignored and falsely autoverified. Under these circumstances, an experienced technologists may identify errors by comparing the results from related tests. For example, the L index is an indicator of the TG level in blood samples. A low, but not extreme, TG result with a high L index suggests that the measurement of TG was faulty, and that the sample should be diluted (Rule 3, TG/L index). Similarly, CRE and UREA are both increased in cases of kidney injury. A low CRE result accompanied by a high UREA result suggests a false CRE measurement, which is often because the CRE test has limited tolerance for drug interference (Rule 4, CRE/UREA). In this situation, the sample should be re‐measured by another enzymatic method or by a dry‐slide method. Therefore, we added the nine consistency check rules to the workflow (Figure 1). In our experience, violation of Rule 1 or 2 ((IgG + IgM + IgA)/(KAP + LAM), or (IgG + IgM + IgA)/(TP − ALB) suggests the hook effect in one or more tests.^10^, ^11^ Substrate depletion is an important reason for violation of Rules 5, 6, and 7 (ALT/AST, TBIL/DBIL, ALP/γ‐GT), while aging of electrodes is for violation of Rule 8 (NA/CL, the two ions usually measured by electrodes). Moreover, violation of Rule 9 (CA × P) implies sample aspiration error or a lower CA induced by EDTA‐K_2_.

Since the follow‐up actions for rule violations vary, the preset priority of autoverification rules helps laboratory technologists to determine which response is needed. Rules with a higher priority should be taken more seriously (Figure 1). Violation of logical rules is unacceptable, and the intercepted report cannot be verified, even manually. Hence, all the factors in the tests, including specimen characteristics, instrument conditions, and quality controls, should be checked to identify the problem, and after the problem has been resolved, the test should be re‐run. If a HIL index, particularly the H index, exceeds the extreme value range, the sample quality may not meet the requirements for some tests and re‐sampling is needed. Violation of consistency check rules suggests that the test conditions may not fit the specimen. If the technologists find that the specimen or the reaction curve is abnormal, dilution of the specimen or an alternative methodology is recommended; otherwise, the report is manually verified. For reports violating limit check or delta check rules, the patient information and medical history should be reviewed to determine if the test result is supported. If it is supported, the report is manually verified; otherwise, the test should be repeated to verify that the result is true.

The classic knowledge‐based range‐determining strategy for autoverification systems has faced challenges. It takes time for laboratory technologists to gain enough verification experience for a newly developed clinical chemistry test. If the instrument or the reagents are upgraded, the previous experience may not be applicable. Regional differences also restrict the application of the autoverification ranges established by one hospital to another hospital in a different region. Moreover, due to better understanding of pathophysiology and laboratory tests, increasingly complex rules have been introduced into the autoverification systems, such as the delta check and consistency check rules. It is difficult for the laboratory technologists to determine the ranges for these rules based on their experience.^12^, ^13^, ^14^, ^15^, ^16^, ^17^ The key cause of these challenges is the slow generation of the quantitative experience (autoverification ranges) from the vast amount of historical clinical report data. More specifically, although the production of the historical clinical report data is rapid, the transformation rate by humans (from data to experience) is limited. This study showed that analysis of historical data by computer expedited this transformation. Moreover, efficiency was not affected by the complexity of the rules (Figures 2 and 3). The effectiveness of the improved autoverification system was competitive and the pass rate (efficiency) increased, which indicates that it is both feasible and practical to use a historical data percentile‐based range‐determining strategy (Table 3).

The consistent increases in the pass rates among the 20 laboratories and the further increase with the customized system indicates that Zhongshan framework did not reverse the efficiency outside Zhongshan Hospital. The historical data percentile‐based range‐determining strategy made the knowledge‐based Zhongshan framework widely applicable (Figure 4). To examine the improved system outside of Zhongshan Hospital, the clinical chemistry tests at other laboratories were all analyzed by similar instruments. In fact, the construction of the framework relied on medical knowledge and determination of the criteria depended on historical data, rather than being restricted to specific instruments. Therefore, even if transferability of the improved autoverification system with the Zhongshan criteria was limited, the Zhongshan framework and the combined strategy to generate an autoverification system was still transferrable.

This study had some limitations. For example, the frequency distributions of different tests/rules vary, and the percentile may need to be optimized for each of them. Optimization of the autoverification system should be explored in future studies.

Taken together, our study illustrates how to improve and popularize an autoverification system using a combined strategy of knowledge‐based rule selection and historical data percentile‐based range determination. The demand for autoverification is increasing, but the experience with autoverification is insufficient for many laboratories. Knowledge‐based rule selection allows an experienced laboratory to construct a comprehensive autoverification system with a follow‐up plan. Additionally, use of historical data percentile‐base range determination makes it possible to transfer the system framework to another laboratory. Such an improved autoverification system may help to reduce the proportion of manual audits, improve the turnaround time, and identify false laboratory reports.

## CONFLICT OF INTEREST

The funding organization(s) played no role in the study design; in the collection, analysis, and interpretation of data; in the writing of the report; or in the decision to submit the report for publication. All the authors declare that there is no conflict of interest.

## AUTHOR CONTRIBUTIONS

All the authors have accepted responsibility for the entire content of this submitted article and approved submission.

## Supporting information

Supplementary Material

## Data Availability

The data that supports the findings of this study are available in the supplementary material of this article.
