# Supplementary material for: Combined strategy of knowledge‐based rule selection and historical data percentile‐based range determination to improve an autoverification system for clinical chemistry test results
Source: J Clin Lab Anal. 2022 Jan 10;36(2):e24233. doi: 10.1002/jcla.24233 (PMC8841182; doi:10.1002/jcla.24233)

Table S1. Tests and rules in the autoverification system.

Parameter	Number
Test (details listed in Supplemental Table 3)	
Liver function	11
Kidney function	4
Lipid	9
Immune globulin	15
Enzyme	6
Electrolyte	8
HIL index	3
Other	4
Original rule	
Instrument analytical range	120 (2 for each test, upper limit and lower limit)
Instrument alarm	107
Critical value	8 (4 tests, CA, NA, K, GLU; 2 for each test, upper limit and lower limit)
Logic among tests	4 (listed in Table 1)
Limit check	60 (1 for each test)
Delta check	60 (1 for each test)
Newly added rule	
Extreme value	60 (1 for each test)
Consistency check	9 (listed in Table 1)

Fig. S1. The framework of the original clinical chemistry autoverification system.

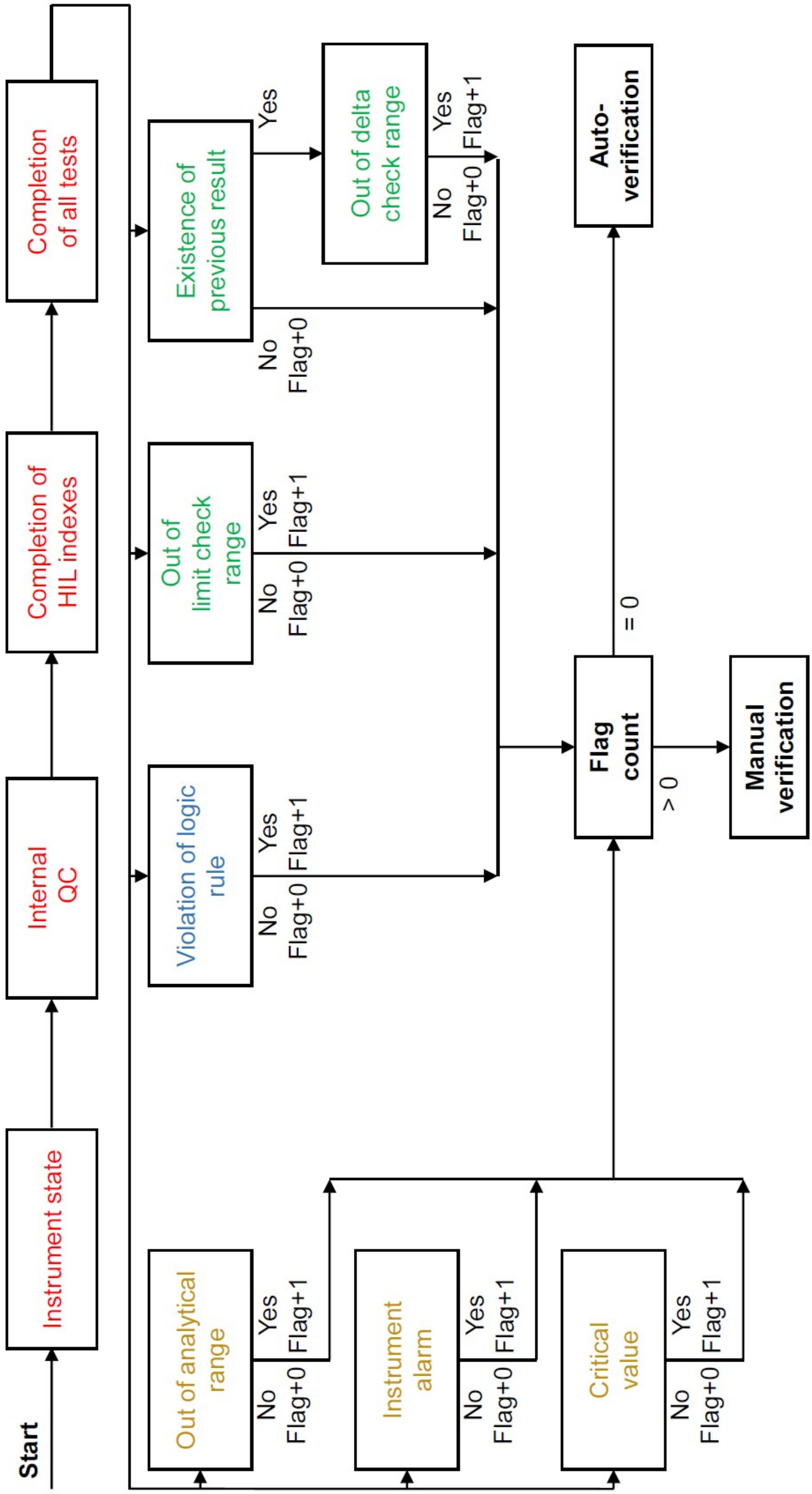

Table S2. The basic information of the multiple centers. The order of the centers in Figure 4 were labeled in red.

District of China	Province	Hospital	Beds (n)	Specimens in 2018 (n, million)	Clinical reports (n)	Validated reports (n)
Eastern	Shanghai	Fudan University Zhongshan Hospital (No 2)	2005	8.0	768119	2506
		Renji Hospital Shanghai Jiaotong University School of Medicine (No 4)	1400	8.0	109481	1704
		Tenth Peoples Hospital of Tongji University (No 8)	1400	2.8	82368	705
		Shanghai DongFang Hospital (No 7)	1800	3.0	71160	555
		Shanghai Songjiang District Central Hospital (No 1)	800	2.3	55437	372
	Jiangsu	The Second Affiliated Hospital of Soochow University (No 3)	2050	3.5	53695	1704
Northern	Beijing	Cancer Hospital Chinese Academy of Medical Sciences (No 20)	1290	1.0	132150	374
		Chinese People's Liberation Army Rocket General Hospital (No 14)	1080	4.0	43083	296
	Tianjing	Tianjin First Central Hospital (No 19)	1500	2.9	1728	270
	Henan	The First Affiliated Hospital of Zhengzhou University (No 13)	7000	4.8	71617	968
	Shandong	Qilu Hospital of Shandong University (No 10)	4300	2.8	51645	3077
	Inner Mongolia	Inner Mongolia People's Hospital (No 16)	2900	2.1	46461	2271
Southern	Guangzhou	The First Affiliated Hospital of Guangzhou University of Chinese Medicine (No 17)	2200	2.5	206880	1480
		Shenzhen People's Hospital (No 11)	2500	3.3	67705	964
		Guangdong Provincial TCM Hospital (No 18)	1000	2.4	96515	680
	Hubei	Tongji Medical College Huazhong University of Science and Technology (No 5)	6000	4.0	75885	298
	Fujian	Zhongshan Hospital Xiamen University (No 9)	1200	1.5	25945	538
Western	Shaanxi	Xijing Hospital (No 15)	2580	4.0	56042	265
	Chongqing	The Hospital Group of The First Affiliated Hospital Of Chongqing Medical University (No 6)	3200	3.5	90472	1426
	Yunnan	First Affiliated Hospital of Kunming Medical University (No 12)	4000	2.5	140309	1097

Fig. S2. The workflow of the validation process. The pooled clinical chemistry reports were manually judged by technologists and an expert panel. The autoverification system judged if each manually verified report passed or failed. The judgment consistence between manual verification an autoverification were used to define “true negative”, “false negative”, “false positive” and “true positive”, respectively.

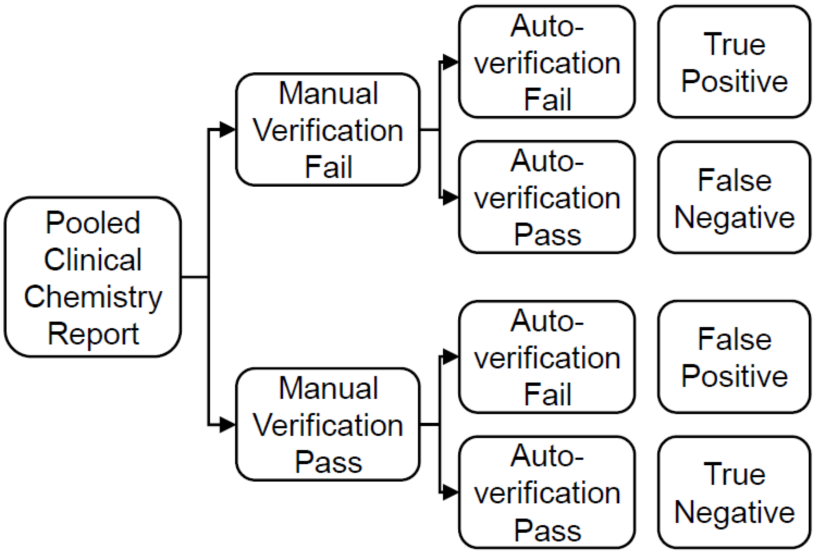

Table S3. The extreme value range, limit check range and delta check range for each test.

No	Test	Full name	Unit	Extreme value range	Limit check range	Delta check range
Liver function						
1	TBIL	Total bilirubin	μmol/L	1.8 - 409.2	3.7 - 45.4	-50% - +132%
2	DBIL	Direct bilirubin	μmol/L	0.3 - 341.9	1.1 - 27.2	-52% - +159%
3	TP	Total protein	g/L	38 - 89	51 - 79	-18% - +15%
4	ALB	Albumin	g/L	19 - 51	29 - 48	-18% - +17%
5	ALT	Alanine aminotransferase	U/L	2 - 1159	6 - 152	-57% - +285%
6	AST	Aspartate aminotransferase	U/L	6 - 1184	11 - 122	-63% - +248%
7	ALP	Alkaline phosphatase	U/L	24 - 1057	39 - 267	-31% - +50%
8	γ-GT	L-γ-Glutamyl transferase	U/L	6.4 - 41.9	10 - 28	-42% - +131%
9	LDH	Lactate dehydrogenase	U/L	0 - 2695	131 - 518	-34% - +78%
10	TBA	Total bile acid	μmol/L	0.1 - 367.7	1 - 57	-88% - +416%
11	PA	Prealbumin	g/L	0.04 - 0.55	0.08 - 0.39	-42% - +48%
Kidney function						
12	CRE	Creatinine	μmol/L	24 - 1476	42 - 596	-30% - +37%
13	UA	Uric acid	μmol/L	50 - 841	110 - 547	-49% - +56%
14	UREA	Urea	μmol/L	1.3 - 46.9	2.5 - 23.7	-48% - +80%
15	CysC	Cystatin C	mg/L	0.36 - 8.69	0.54 - 3.53	-35% - +58%
Lipid						
16	TC	Total cholesterol	mmol/L	1.65 - 11.96	2.54 - 7.07	-35% - +38%
17	TG	Triglyceride	mmol/L	0.3 - 13.8	0.51 - 4.43	-54% - +117%
18	HDL	High density lipoprotein	mmol/L	0.15 - 3.22	0.62 - 2.25	-37% - +57%
19	LDL	Low density lipoprotein	mmol/L	0.44 - 8.53	1.21 - 5.14	-45% - +60%
20	Apo A-I	Apolipoprotein A-I	g/L	0.28 - 2.86	0.79 - 2.22	-33% - +43%
21	Apo B	Apolipoprotein B	g/L	0.31 - 2.25	0.47 - 1.41	-35% - +37%
22	Apo E	Apolipoprotein E	mg/L	11 - 218	21 - 90	-36% - +51%
23	Lpa	Lipoprotein A	mg/L	4 - 1875	13 - 932	-52% - +136%
24	FFA	Free fatty acid	mmol/L	0.05 - 1.05	0.11 - 0.85	-72% - +174%
Immune globulin						
25	IgG	Immunoglobulin G	g/L	2.06 - 72.45	4.74 - 25.1	-28% - +28%
26	IgA	Immunoglobulin A	g/L	0.04 - 15.6	0.24 - 5.52	-29% - +28%
27	IgM	Immunoglobulin M	g/L	0.04 - 24.26	0.18 - 3.24	-32% - +26%
28	IgE	Immunoglobulin E	g/L	0 - 14117	0 - 1691	-70% - +128%
29	RF	Rheumatoid factor	IU/ml	0 - 507	0 - 189	-100% - +50%
30	TRF	Transferrin	g/L	0.6 - 4.89	1.1 - 3.8	-26% - +31%
31	ASO	Anti-streptolysin O	IU/ml	0 - 1035	5 - 298	-50% - +68%
32	KAP	Immunoglobulins of the kappa light chain type	g/L	0.03 - 29.2	0.39 - 7.61	-40% - +62%

Table S3. The extreme value range, limit check range and delta check range for each test (continued).

No	Test	Full name	Unit	Extreme value range	Limit check range	Delta check range
Immune globulin						
33	LAM	Immunoglobulins of the lambda light chain type	g/L	0.04 - 11.99	0.32 - 4.78	-33% - +40%
34	CER	Ceruloplasmin	g/L	0.01 - 0.58	0.13 - 0.45	-50% - +50%
35	β2M	β2 microglobulin	mg/L	2.2 - 15.6	3.3 - 8.1	-58% - +120%
36	C3	Complement C3	g/L	0.04 - 1.91	0.49 - 1.46	-28% - +32%
37	C4	Complement C4	g/L	0.01 - 0.51	0.07 - 0.36	-36% - +50%
38	CH50	Complement hemolysis 50%	U/ml	5 - 85.6	23.1 - 78.8	-30% - +57%
39	CRP	C reactive protein	mg/L	0 - 307.3	0.3 - 163.5	-88% - +2272%
Enzyme						
40	AMY	Amylase	U/L	9 - 1822	26 - 292	-78% - +158%
41	LIP	Lipase	U/L	2.6 - 5419	11 - 470.7	-83% - +250%
42	CHE	Cholinesterase	U/L	800 - 18064	2004 - 12182	-28% - +23%
43	CK	Creatine kinase	U/L	9 - 6735	22 - 705	-80% - +744%
44	CK-MB	Creatine kinase-MB	U/L	3 - 260	7 - 45	-57% - +167%
45	ACP	Acid phosphatase	U/L	3.1 - 6	2.6 - 16.7	-50% - +50%
Electrolyte						
46	NA	Sodium	mmol/L	123 - 160	132 - 145	-6 - +5
47	K	Potassium	mmol/L	2.0 - 6.5	3.1 - 5.1	-0.9 - +0.8
48	CL	Chlorides	mmol/L	83 - 123	92 - 108	-7 - +5
49	CA	Calcium	mmol/L	1.43 - 3.04	1.87 - 2.54	-0.3 - +0.1
50	P	Inorganic phosphorus	mmol/L	0.23 - 3.82	0.53 - 2.27	-51% - +72%
51	MG	Magnesium	mmol/L	0.31 - 1.47	0.64 - 1.12	-20% - +21%
52	TCO2	Total carbon dioxide	mmol/L	11.6 - 37.8	17.7 - 30.6	-19% - +23%
53	AG	Anion gap	mmol/L	6 - 30	9 - 21	-6 - +5
HIL index						
54	H index	Hemolysis index		0 - 213	1 - 51	-92% - +492%
55	L index	Lipemia index		3 - 599	8 - 118	-69% - +188%
56	I index	Icterus index		3 - 629	10 - 67	-47% - +88%
Other						
57	GLU	Fasting glucose	mmol/L	2.4 - 22	4 - 12.2	-40% - +67%
58	FE	Iron	μmol/L	0.5 - 60.4	1.9 - 27.9	-53% - +144%
59	UIBC	Unsaturated iron binding capacity	μmol/L	1 - 95	9 - 78	-42% - +85%
60	HCY	Homocysteine	μmol/L	3.5 - 190	5.5 - 47.3	-59% - +104%

Fig. S3. The pass rates of 20 laboratories. The data from 20 laboratories were processed using the original (before improvement) and improved (after improvement) autoverification system. For the laboratory (No. 20) with the lowest pass rate, the autoverification system was further improved by establishing the delta check ranges using their own clinical results (Zhongshan framework and partially-customized criteria) and the new pass rate (further improvement) was shown in red.

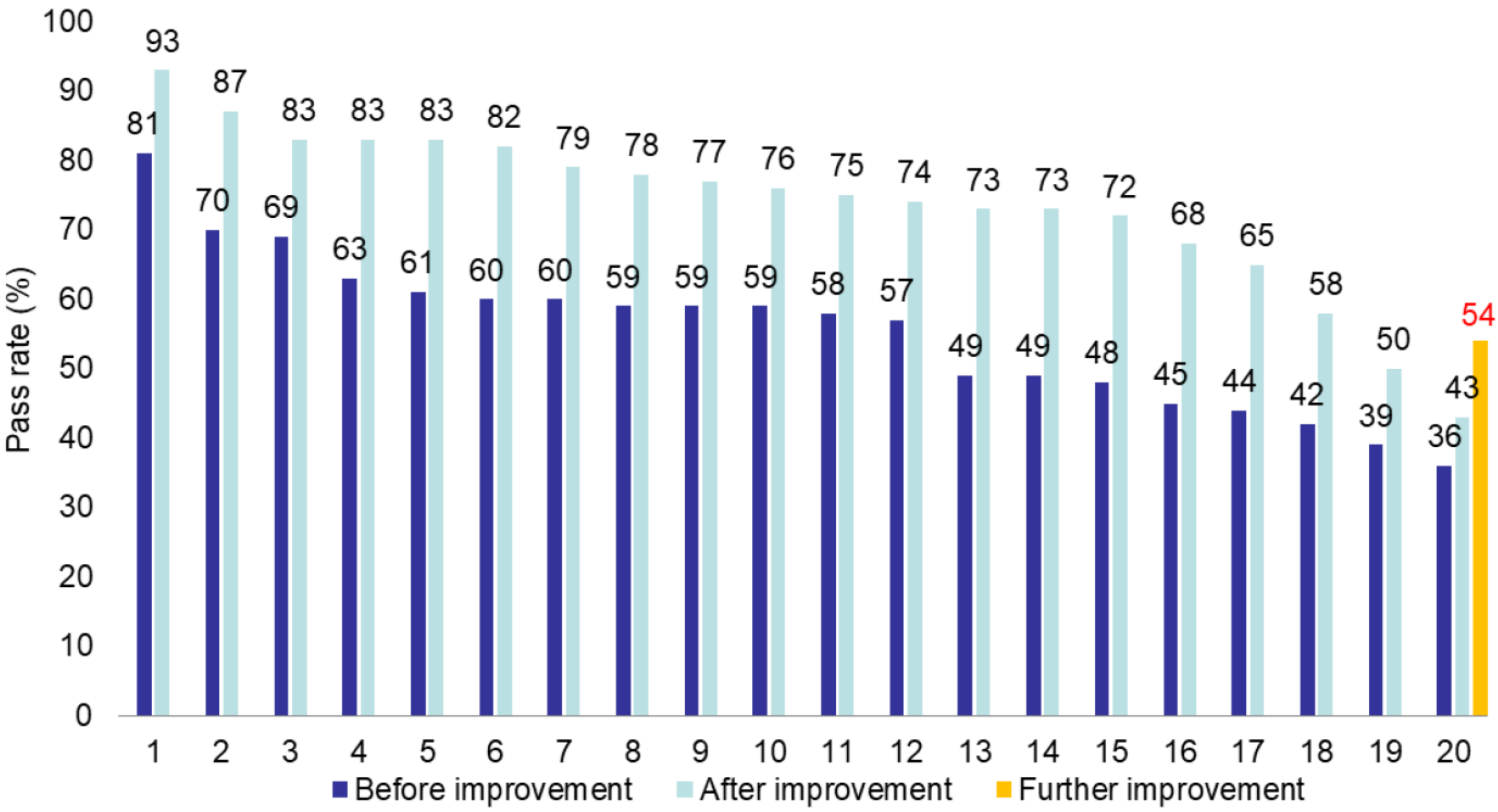

Supplement: Supplementary file 1 [file JCLA-36-e24233-s001.docx]
